# Superb microvascular imaging for evaluating the activity of juvenile localised scleroderma: a preliminary study

**DOI:** 10.1007/s00330-024-10738-z

**Published:** 2024-04-23

**Authors:** Xiaoyi Chen, Luyao Zhou, Yu Xia, Yik Ning Wong, Qiancheng He, Pengyue Tang, Shuangshuang Zhang, Tingting Liu, Ziyi Wang, Na Xu

**Affiliations:** 1 Department of Ultrasound, Shenzhen Children’s Hospital of China Medical University, Shenzhen, 518000 China; 2 Department of Ultrasound, Shenzhen Pediatrics Institute of Shantou University Medical College, Shenzhen, 518000 China; 3 Department of Rheumatology and Immunology, Shenzhen Pediatrics Institute of Shantou University Medical College, Shenzhen, China; 4 Canon Medical Systems (China) Co. Ltd., Beijing, China; 5 Department of Dermatology, Shenzhen Pediatrics Institute of Shantou University Medical College, Shenzhen, China

**Keywords:** Scleroderma, Localised, Activity, Skin, Ultrasonography

## Abstract

**Objectives:**

To investigate microvascular changes in juvenile localised scleroderma (JLS) lesions using superb microvascular imaging (SMI) and assess SMI’s utility in evaluating disease activity.

**Methods:**

This prospective study enroled 16 children (7 males) with pathologically diagnosed JLS between January 2021 and June 2023. Lesions were assessed using Localised Scleroderma Cutaneous Assessment Tools, including the localised scleroderma skin activity index (LoSAI) and localised scleroderma skin damage index (LoSDI). Lesions with LoSAI scores > 0 were classified as active. The thickness and blood flow of the lesions and healthy skin layers of the contralateral site were evaluated using ultrasound. SMI was used to detect microvascular blood flow in the lesions and healthy skin, and the vascular index (VI) was calculated. The difference in VI between active lesions and healthy skin was correlated with LoSAI and total scores.

**Results:**

Of 46 lesions, 23 were active and 23 inactive. The skin thickness of the lesion was 0.094 ± 0.024 cm, and that of the healthy site was 0.108 ± 0.026 cm (*p* < 0.001). The VI of the active lesions and healthy skin were 7.60 (3.60, 12.80)% and 1.10 (0.50, 2.10)%, respectively (*p* < 0.001). The VI of the inactive lesions and the healthy skin were 0.85 (0.00, 2.20)% and 1.60 (1.00, 3.10)%, respectively (*p* = 0.011). VI differences between active lesions and healthy skin positively correlated with the LoSAI clinical score (*r* = 0.625, *p* = 0.001) and total score (*r* = 0.842, *p* < 0.001).

**Conclusion:**

SMI can quantitatively detect microvascular blood flow changes in JLS skin, indicating lesion activity and severity.

**Clinical relevance statement:**

SMI is a convenient, non-invasive, technique for detecting active JLS lesions and can provide valuable information to guide treatment options.

**Key Points:**

- *Current grading systems of juvenile localised scleroderma rely on subjective clinical information*.
- *Superb Microvascular Imaging identified that vascular indexes between active lesions and healthy skin positively correlated with clinical scores*.
- *Superb Microvascular Imaging effectively assesses microvascular blood flow, aiding juvenile localised scleroderma lesion activity evaluation*.

## Introduction

Scleroderma is a chronic connective tissue disease categorised by a wide range of microvascular damage in the skin and internal organs as well as excessive collagen deposition. Scleroderma is categorised into systemic and localised scleroderma (LS) [1–3], with the latter being the most frequently diagnosed type in children. Pathologically, LS can be classified into inflammatory (active), sclerotic, and atrophic stages. In the early stages, inflammatory cells infiltrate blood vessels leading to structural vascular changes and neovascularisation. As the inflammation subsides, collagen fibres accumulate resulting in homogenisation of subcutaneous tissues [4]. Concurrently, blood vessels become thinner and narrower [5, 6], causing damage to the skin and multiple organs. While LS primarily affects the skin, it can also involve adipose tissue, muscles, and fascia [7]. In severe cases, it can lead to limb movement disorders, facial or limb deformities, and motor function disorders in children. Unlike in adults, the occurrence and development of localised scleroderma in children is occult. The activity and severity of the lesions lead to different treatment methods. Topical treatment can be used in active lesions with single or small lesions, while in severe cases methotrexate is preferred [8].

In 2012, the Childhood Arthritis And Rheumatology Research Alliance (CARRA) published evaluation criteria for juvenile localised scleroderma (JLS) disease activity and related clinical evaluation parameters [9]. Localised scleroderma cutaneous assessment tools (LoSCATs) are commonly used to clinically assess LS [10]. However, these tools lack objective and quantitative indicators. Meanwhile, changes in soft tissue, bone, joint tendons, and blood vessels can be detected through imaging [11]. Conventional ultrasound and ultrasound elastography have been used to assess JLS lesion thickness, scope, blood vessels, and hardness [12–14]. Nevertheless, it is still challenging to precisely evaluate JLS with traditional ultrasound techniques.

Superb microvascular imaging (SMI) is an innovative vascular Doppler imaging technology capable of visualising low-velocity blood flow while remaining unaffected by motion artefacts from nearby structures [15]. Compared to colour Doppler technology, SMI exhibits heightened sensitivity for microvasculature [16]. SMI is primarily used for evaluating thyroid, gynaecological, breast, liver, and kidney conditions [17–21], offering valuable insights into angiogenesis-related diseases to support diagnosis and treatment. The skin and subcutaneous tissues are rich in capillaries with small diameters and slow blood flow rates. In early-stage lesions, inflammation causes blood vessels to become tortuous. Therefore, this study aimed to investigate microvascular changes in JLS lesions using SMI and to assess SMI’s utility in evaluating disease activity.

## Methods

### Study population

This study was approved by the Ethics Committee of Shenzhen Children’s Hospital (Approval number: 2022043). Written informed consent was provided by the participants’ legal guardians or next of kin.

This was a prospective study. Sixteen children pathologically diagnosed with LS at either our dermatology or rheumatology departments between January 2021 and June 2023 were included. Inclusion criteria were: diagnosis of JLS confirmed by skin biopsy, age < 18 years, and complete clinical medical records. Exclusion criteria were systemic sclerosis, juvenile rheumatoid arthritis, other skin diseases, and incomplete clinical data.

### Ultrasound examination

The Aplio i900 ultrasonic diagnostic instrument (CANON Aplio i900, Canon Medical Systems Corporation, Japan) and i24LX8 linear probe were used for skin imaging. All ultrasound examinations were performed by a sonographer with 10 years of experience and finishing the SMI training in Canon machine operation. The active lesion was marked by a doctor of the rheumatology department with 16 years of experience, and relatively normal skin was marked on the opposite side (with no erythema, atrophy, and dyspigmentation).

**Fig. 1:**
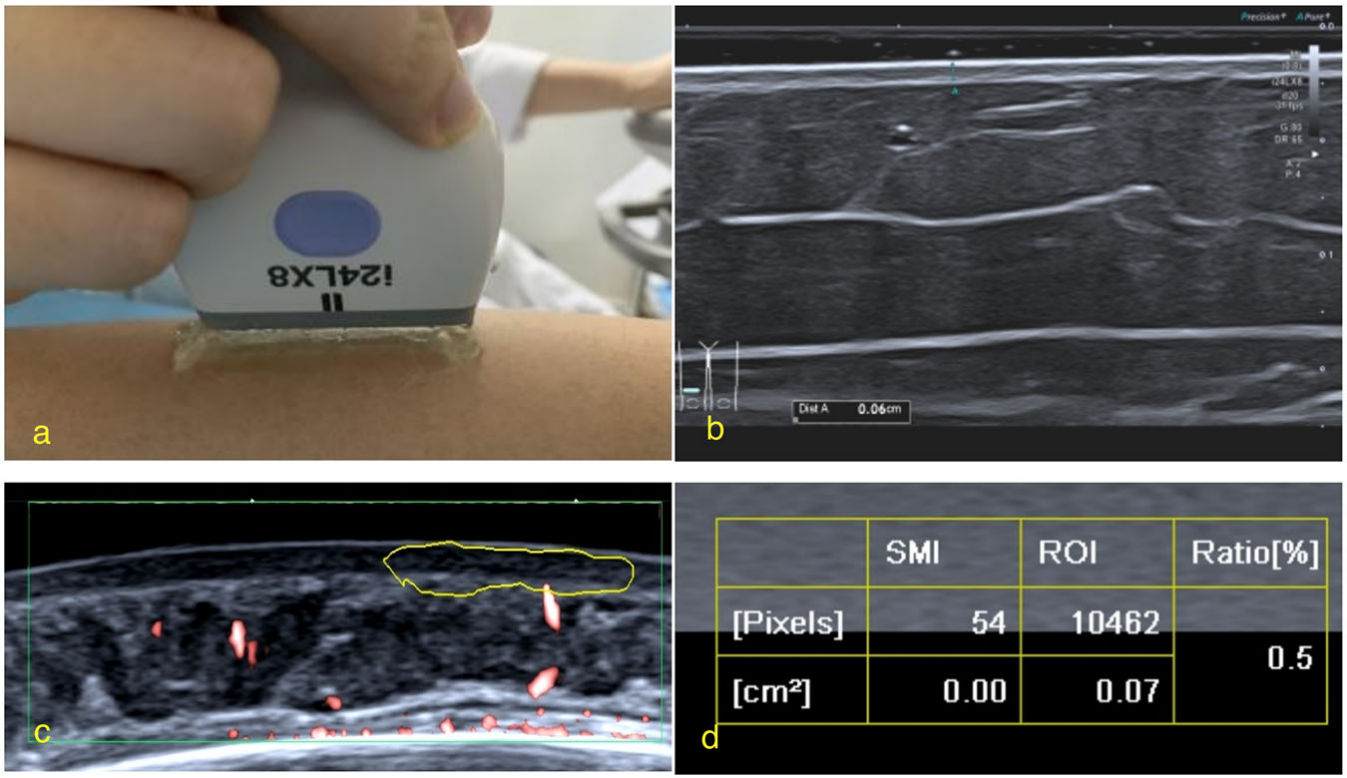
Ultrasonic examination and measurement. **a** The ultrasonic probe was placed vertically without exerting pressure or direct skin contact. **b** Measurement map of skin layer thickness on the right thigh. **c** Vascular index (VI) measurement image of microvascular blood flow in the skin layer for normal skin on the left forehead. **d** The manually measured pixel value in the region of interest (ROI) was 10462, and the VI was 0.5%

Participants were positioned comfortably, and a uniform layer of gel was applied to the probe. The probe was placed gently on the skin, perpendicular to the skin level. Two-dimensional ultrasound images of the skin and subcutaneous tissue of the lesions and normal contralateral sites were obtained, and the thickness of the skin layer was measured vertically after zooming in. Colour Doppler and SMI were performed on the skin layer of the JLS lesions and the healthy skin layer of the same contralateral site. Appropriate sampling frames were selected based on lesion size, and all selected areas were scanned. Low-range blood flow conditions were chosen for the colour Doppler, and the colour gain was adjusted to no noise signal. Skin thickness and SMI measurements were performed at the same location. Vascular index (VI), a ratio that the blood flow pixel value of measurement area accounts for the whole region of interest pixel (Fig. 1c), was manually measured with the most abundant blood flow area in the target region. The pixel value of the skin layer measurement was limited to 9000–11,000. The difference in the areas of interest measured on both sites was less than 1000 pixels (Fig. 1). Two doctors with more than 5 years of experience separately measured each site twice and averaged the two measurements. The final thickness of the skin layer and the VI value corresponded to the average of the two doctors’ values.

### Clinical assessment of lesion activity

The activity of JLS lesions was assessed by an experienced rheumatologist using the clinical scores of the localised scleroderma skin activity index (LoSAI) (Table 1) and localised scleroderma skin damage index (LoSDI) (Table 2) for each lesion. The total score was calculated by summing the LoSAI score and the LoSDI score, and lesions were considered to be in the active stage if the LoSAI score was greater than 0 [22].

**Table 1:** Modified localised scleroderma skin severity index

New lesions/enlarged (past month)	Erythema	Skin thickness
0 = none	0 = none	0 = none
	1 = slight, pink	1 = slight increase
3 = new lesions/enlarged	2 = clear, red	2 = moderate increase, difficulty moving skin
	3 = marked, dark red	3 = marked increase, skin cannot move

**Table 2:** Localised scleroderma skin damage index

Dermal atrophy	Subcutaneous atrophy	Dyspigmentation
0 = none	0 = none	0 = none
1 = slight	1 = flat	1 = slight
2 = moderate, visible blood vessel	2 = concave	2 = moderate
3 = severe, depresses easily when pressed	3 = marked	3 = marked

### Statistical methods

Statistical analysis was performed using SPSS version 26.0 0 (SPSS, Chicago, IL, USA). Normally distributed data were presented as mean ± SD. Non-normally distributed data were expressed as medians along with their lower and upper quartiles. Skin thickness of the lesion and healthy sites were compared using a paired sample *t*-test. The Wilcoxon rank-sum test was used to compare the VI between active lesions and healthy skin. The VI difference in microvascular blood flow between active lesions and the healthy skin layer was calculated. The correlation between the VI difference and LoSAI score, as well as the total score, was analysed using Spearman’s correlation analysis. Regarding correlation coefficients (*r*), *r* < 0.10 indicated a negligible correlation, 0.10–0.39 a weak correlation, 0.40–0.69 a moderate correlation, 0.70–0.89 a strong correlation, and ≥ 0.90 a very strong correlation. Statistical significance was set at *p* < 0.05. The intraclass correlation coefficient (ICC) was used to investigate the consistency between the two doctors’ measurements of the lesions and healthy skin. Intra-observer ICC estimates were calculated using SPSS based on a single rater, consistency, two-way mixed-effects model. Inter-observer ICC estimates were calculated using SPSS based on a multiple raters (*k* = 2), absolute-agreement, two-way random-effects model. ICC < 0.40 indicated a poor agreement, 0.40–0.54 a weak agreement, 0.55–0.69 a moderate agreement, 0.70–0.84 a good agreement, and 0.85–1.00 an excellent agreement.

## Results

### Clinical scores of lesion sites

The demographic and clinical characteristics of the 16 participants (7 males) are summarised in Table 3, with a median age of 8 (5, 10) years and a median course of disease of 3 (2, 4) years. A total of 46 lesions were located on the face (*n* = 12), upper limbs (*n* = 5), chest (*n* = 2), abdomen (*n* = 6), back (*n* = 2), and lower limbs (*n* = 19). According to the LoSAI scoring tool, 23 lesions scored > 0 points (Table 4) and were active, whereas 23 lesions scored 0 points and were inactive.

**Table 3:** Demographic and clinical characteristics of participants

Patient characteristics		Numerical value
Sex (*n* = 16)	female	9 (56%)
	male	7 (44%)
Age (years)		8 (5, 10)
Disease duration (years)		3 (2, 4)
Lesions (*n* = 46)	active	23 (50%)
	inactive	23 (50%)
Lesion body parts (*n* = 46)	face	12 (26%)
	upper limbs	5 (11%)
	chest	2 (4%)
	abdomen	6 (13%)
	back	2 (4%)
	lower limbs	19 (41%)

**Table 4:** LoSAI and total scores of 23 juvenile localised scleroderma active lesions

Patients ID	Lesion part	LoSAI	LoSDI	Total
01	forehead	4	1	5
02	left thigh	3	0	3
	chest	5	0	5
03	face	1	0	1
04	face	5	4	9
	forehead	2	2	4
05	left thigh	4	1	5
06	abdomen	3	0	3
07	face	4	2	6
08	back	4	0	4
10	left foot	2	0	2
11	left thigh	5	1	6
12	chest	2	2	4
	right arm	4	4	8
	right hand	5	1	6
	left hand	1	0	1
13	abdomen	2	0	2
	chest	4	0	4
14	forehead	1	4	5
	face	4	1	5
15	left leg	1	3	4
16	scalp	2	1	3
	forehead	1	4	5

None of the Patient 09’s lesions were active

*LoSAI* localised scleroderma skin severity index, *LoSDI* localised scleroderma skin damage index, *ID* identification

### Skin thickness difference between the lesion and healthy sites

The skin thickness at 46 lesion sites was 0.094 ± 0.024 cm, while the skin thickness at healthy sites was 0.108 ± 0.026 cm, and the difference was statistically significant (*p* < 0.001).

### Colour doppler and VI results for skin layer in the lesion and healthy sites

**Fig. 2:**
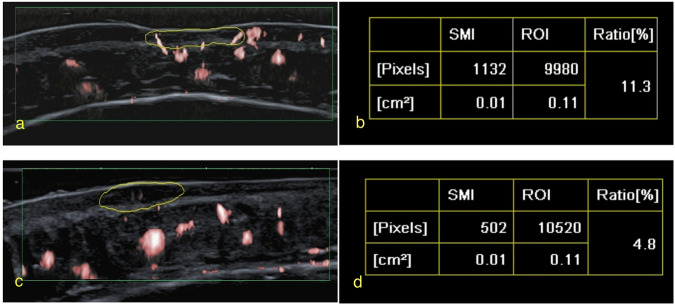
A 9-year-old boy with pathologically confirmed juvenile localised scleroderma. The disease duration was 2 years. Over the previous month, the lesions in the middle of the forehead showed enlargement, slight erythema, and slight downward depression. **a** Superb microvascular imaging (SMI) image of the active lesion skin layer in the middle of the forehead. **b** The measured Vascular index VI was 11.3%. **c** SMI image of healthy skin layer on the right side of the forehead. **d** The measured VI was 4.8%

**Fig. 3:**
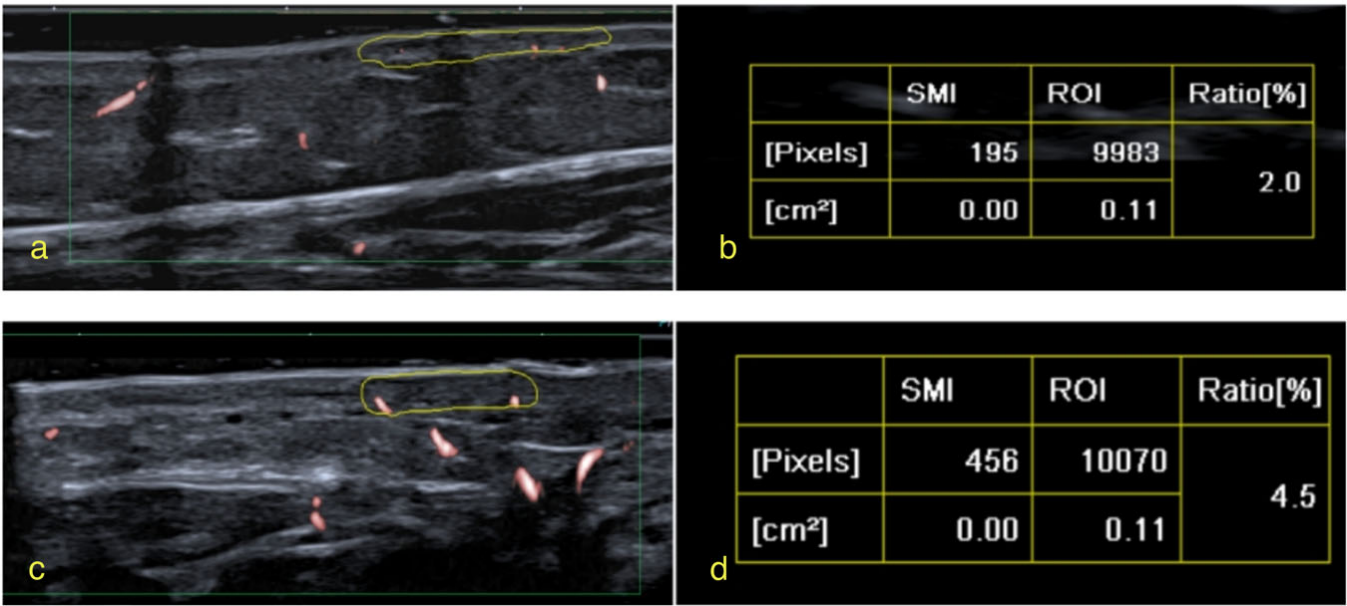
An 8-year-old girl with pathologically confirmed juvenile localised scleroderma. The disease duration was 2 years. The boundary between the skin and the subcutaneous tissue of the inactive lesion of the left thigh was unclear. **a** Superb microvascular imaging (SMI) image of the inactive diseased skin layer of the left thigh. **b** The measured vascular index (VI) was 2.0%. **c** SMI image of the healthy skin layer on the right thigh. **d** The measured VI was 4.5%

**Fig. 4:**
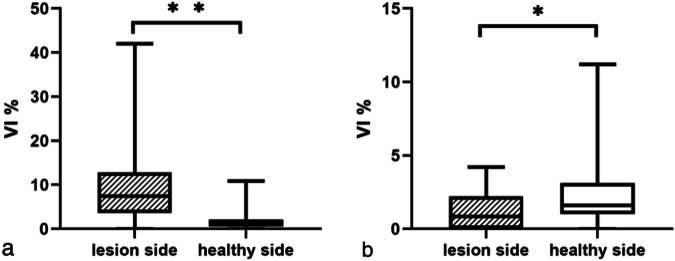
Comparison of vascular index (VI) between juvenile localised scleroderma (JLS) lesion and healthy sites. **a** Comparison of VI between JLS active lesions and healthy sites. **b** Comparison of VI between JLS inactive lesions and healthy sites. *p < 0.05; **p < 0.001

In 46 lesions, colour Doppler could not detect the blood flow in the skin layer, and nine lesions were detected in the deep subcutaneous tissue. The skin layer microvascular flow of 39 lesions could be detected by SMI. The VI of the 23 active JLS lesions was compared to that of the skin at healthy sites. The microvascular blood flow VI of the skin in active lesions and healthy sites was 7.60 (3.60, 12.80)% and 1.10 (0.50, 2.10)%, respectively (*p* < 0.001; Fig. 2). The microvascular blood flow VI of the skin in inactive lesions and healthy sites was 0.85 (0.00, 2.20)% and 1.60 (1.00, 3.10)%, respectively. The VI of the inactive lesions was lower than that of the healthy sites (Fig. 3), and the difference was statistically significant (*p* = 0.011; Fig. 4).

### Correlation analysis of the difference of VI between active lesions and healthy skin layers with LoSAI and total score

**Fig. 5:**
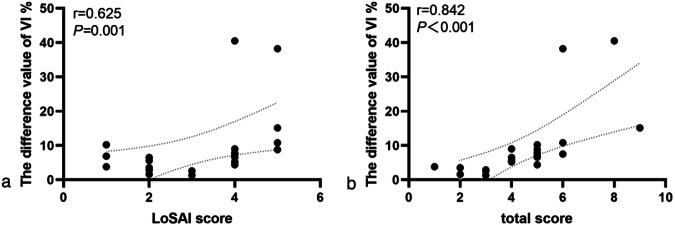
Correlation between the difference value of vascular index (VI) calculated for the active lesions and the healthy sites and the localised scleroderma skin activity index (LoSAI) and total scores. **a** Correlation between the difference value of VI calculated for the active lesions and the healthy sites and the LoSAI scores. **b** Correlation between the difference value of VI calculated for the active lesions and the healthy sites and the total scores

The difference in VI between active lesions and healthy skin microvascular blood flow was positively correlated with the LoSAI score (*r* = 0.625, *p* = 0.001). The higher the LoSAI score, the larger the VI difference. It was also positively correlated with the total score (*r* = 0.842, *p* < 0.001); the higher the total score, the larger the VI difference (Fig. 5).

### Repeatability test

The skin thickness and VI measurements were performed in 46 lesions and healthy sites twice by two doctors. The consistency of each doctor’s measurements was good. The ICC values for Doctor 1’s twice measurements were 0.97, 0.97, 0.99, and 0.98, respectively. The ICC values for Doctor 2’s twice measurements were 0.93, 0.97, 0.99, and 0.99, respectively. The consistency between the average values of the two doctors’ measurements was good, with ICC values of 0.95, 0.98, 0.97, and 0.99, respectively (Table 5), and all the differences were statistically significant (*p* < 0.001).

**Table 5:** Consistency between the inspection of skin thickness and vascular index (VI) measurements by two doctors

Group	Skin thickness (lesion side) cm	Skin thickness (healthy side) cm	VI (lesion side) %	VI (healthy side) %
Doctor 1 measure 1	0.092 ± 0.025	0.107 ± 0.027	2.75 (1.00, 7.35)	1.55 (0.60, 2.48)
Doctor 1 measure 2	0.091 ± 0.024	0.107 ± 0.026	2.80 (1.15, 8.05)	1.35 (0.58, 3.00)
ICC	0.97	0.97	0.99	0.98
Doctor 2 measure 1	0.093 ± 0.023	0.108 ± 0.027	2.40 (0.78, 7.43)	1.50 (0.60, 2.55)
Doctor 2 measure 2	0.096 ± 0.025	0.107 ± 0.026	2.70 (0.65, 7.53)	1.45 (0.60, 2.85)
ICC	0.93	0.97	0.99	0.99
Doctor 1 average	0.092 ± 0.024	0.107 ± 0.026	2.65 (1.35, 7.50)	1.50 (0.60, 2.63)
Doctor 2 average	0.096 ± 0.025	0.108 ± 0.026	2.60 (0.68, 7.53)	1.50 (0.60, 2.55)
ICC	0.95	0.98	0.97	0.99

## Discussion

In this study, the overall skin thickness of the 46 lesions was thinner than that of healthy sites, a phenomenon potentially linked to disease progression. The median disease duration among the 16 participants was 3 years, marking disease progression into the middle and late stages–categorised by atrophy and fibrosis. Consistent with these results, Perez et al [14] reported that the skin with LS lesions was 30.7% thinner than the corresponding healthy skin, with occasional tissue thickening. However, in our study, seven lesions exhibited greater skin thickness than the healthy sites, with five categorised as active and two as inactive lesions. As noted by Li et al [23], distinguishing active and inactive lesions based solely on skin thickening at the lesion’s edge or centre may be insufficient, warranting consideration of additional factors such as lesion size and colouration.

Colour Doppler was not good at detecting capillaries with small diameters and slow blood flow rates. Among the 46 lesions in our study, blood flow in the skin layer was not visualised by colour Doppler; blood flow from only nine lesions was detected in the deep subcutaneous tissue. Li et al [24] used ultrasonic colour Doppler technology to examine clinically diagnosed LS lesions, reporting increased blood flow signals in the subcutaneous tissues. However, the blood flow in the skin layer was not evaluated. Similarly, Perez et al [14] observed abnormal blood vessel distribution and increased blood flow, relative to that in contralateral vessels, in only three of seven active lesions. Existing studies have shown that colour Doppler imaging of skin layer blood flow is poor, which is similar to our result.

SMI effectively distinguishes low-velocity blood flow from motion artefacts [25]. Currently, laser speckle contrast analysis and SMI have been used to evaluate nailfold blood perfusion in patients with systemic scleroderma [26, 27], confirming the validity of SMI assessment. Thus, compared to colour Doppler, SMI increased the visibility of the low-velocity blood flow findings. The two active lesions that lacked blood flow were considered to have been reactivated from long-term inactive lesions, with skin fibrosis and atrophy attenuating the visibility of microvascular blood flow.

In this study, active lesions had significantly higher VI than healthy skin, aligning with the active phase of pathology. The VI in the skin layer of inactive lesions was lower than that of the healthy sites, indicating that middle and late-stage lesions categorised by skin and subcutaneous tissue fibrosis could reduce blood vessel distribution [28]. Nineteen lesions were located in the lower extremities, most of them being inactive; most of the active lesions were located in the upper body. This may be the reason for the slight difference between the two healthy site values.

The VI difference correlated with clinical scores. Currently, the LoSAI score is mainly used to evaluate the activity and severity of JLS lesions, while the LoSDI score evaluates LS skin injury severity [29]. The severity of JLS lesion activity is related to the LoSAI score, with higher scores indicating more severe activity [22]. However, clinical judgement is subjective, necessitating additional tests. He et al [30] observed that the Young’s modulus of the JLS’ lesion skin was moderately correlated with LoSAI score, but not with LoSDI score. This change in skin hardness may be related to the changes in the skin layer distribution of collagen during the active period. In addition, the difference in VI was more positively correlated with the total score than the LoSAI clinical scores. Considering that the LoSDI score evaluates JLS skin injury, it can also stimulate vascular tortuosity and hyperplasia to some extent, leading to increased blood flow and further alterations in VI.

Due to differences in vascular distribution across age groups and body locations [15], this study did not compare participants with healthy volunteers. Instead, we conducted a comparative study with the healthy skin layer of the same contralateral site.

This study has several limitations. First, the rarity of the disease and the small sample size could introduce bias into the results. Second, the ultrasound SMI examination was performed by a single doctor, introducing subjectivity into the results and potentially affecting the accuracy of blood flow assessments. Finally, there was no follow-up evaluation of the treatment effect on JLS after SMI examination. Future research should address these limitations and further explore the potential applications of SMI in paediatric dermatology to refine diagnostic and treatment approaches. Subsequently, multi-centre cooperation could be conducted to expand the sample size.

In conclusion, this study utilised SMI technology to quantitatively assess microcirculation changes in active lesions of patients with LS. The non-invasive and convenient nature of SMI makes it a valuable tool for assessing disease activity in children with JLS and guiding treatment decisions.

## Data Availability

The datasets generated and/or analysed during the current study are not publicly available because of the regulations of Shenzhen Children’s Hospital and the protection of patient personal information. Data generated or analysed during the study are available from the corresponding author by request.

## Abbreviations

JLS: Juvenile localised scleroderma
LoSAI: Localised scleroderma skin activity index
LoSCAT: Localised scleroderma cutaneous assessment tools
LoSDI: Localised scleroderma skin damage index
SMI: Superb microvascular imaging
VI: Vascular index
